# Supplementary material for: A New Macrodiolide and Two New Polycyclic Chromones from the Fungus Penicillium sp. SCSIO041218
Source: Molecules. 2019 Apr 30;24(9):1686. doi: 10.3390/molecules24091686 (PMC6539008; doi:10.3390/molecules24091686)
Supplement: Supplementary file 1 [file molecules-24-01686-s001.pdf]

A New Macrodiolide and Two New Polycyclic Chromones from the Fungus *Penicillium* sp. SCSIO041218

Jingxia Huang [1,†], Jianglian She [2,†], Xiliang Yang [2,*], Juan Liu [3], Xuefeng Zhou [3] and Bin Yang [3,*]

1. Zhongshan Ophthalmic Center, Sun Yat-Sen University, Guangzhou 510060, China; 13694217880@163.com
2. Department of Pharmacy, Hubei Province Key Laboratory of Occupational Hazard Identification and Control, Institute of Infection, Immunology and Tumor Microenvironments, Medical College, Wuhan University of Science of Technology, Wuhan 430081, China; sjlsjl0210@163.com
3. CAS Key Laboratory of Tropical Marine Bio-resources and Ecology/Guangdong Key Laboratory of Marine Materia Medica, South China Sea Institute of Oceanology, Chinese Academy of Sciences, Guangzhou 510301, China; liujuan@scsio.ac.cn (J.L.); xfzhou@scsio.ac.cn (X.Z.);

* Correspondence: yxlyxl117@163.com (X.Y); yangbin@scsio.ac.cn (B.Y.)

† These two authors contributed equally to this work.

Supporting information

Table of Contents

Biological assays

Figure S1. ^{1}H NMR spectra of **1** in CD_3OD

Figure S2. ^{13}C NMR spectra of **1** in CD_3OD

Figure S3. HSQC spectra of **1**

Figure S4. HMBC spectra of **1**

Figure S5. ^{1}H–^{1}H COSY spectrum of **1**

Figure S6. HRESIMS spectrum of **1**

Figure S7. ^{1}H NMR spectra of **2** in CD_3OD

Figure S8. ^{13}C NMR spectra of **2** in CD_3OD

Figure S9. HSQC spectra of **2**

Figure S10. HMBC spectra of **2**

Figure S11. HRESIMS spectrum of **2**

Figure S12. Compare ^{1}H NMR spectra between **2** and **3**

Figure S13. Compare ^{13}C NMR spectra between **2** and **3**

Figure S14. CD data of **1**

Figure S15. CD data of **2** and **3**

Biological assays

Cytotoxicity was assayed with the CCK8 (DOjinDo, Japan) method [1]. Cells were routinely grown and maintained in RPMI or DMEM with 10% FBS and with 1% penicillin/streptomycin. All cell lines were incubated in a Thermo/ Forma Scientific CO2 water-jacketed incubator with 5% CO2 in the air at 37°C. The cell viability assay was determined by CCK8 (DOjinDo). The cells were seeded at a density of 400–800 cells/well in 384-well plates and treated with various concentrations of compounds or solvent control. After 72 h incubation, CCK8 reagent was added, and absorbance was measured at 450nm using an Envision 2104 multi-label reader (Perkin Elmer). Dose-response curves were plotted to determine the IC_{50} values using Prism 5.0 (GraphPad Software, Inc.). TSA (trichostatin A, purity ≥ 98%; Sigma) was used as the positive control.

References

[1] Wang, J.; Wang, Z.; Ju, Z.; Wan, J.; Liao, S.; Lin, X.; Zhang, T.; Zhou, X.; Chen, H.; Tu, Z.; Liu, Y. Cytotoxic Cytochalasins from Marine-Derived Fungus *Arthrinium arundinis*. *Planta Med.* **2015**, 81, 160–166.

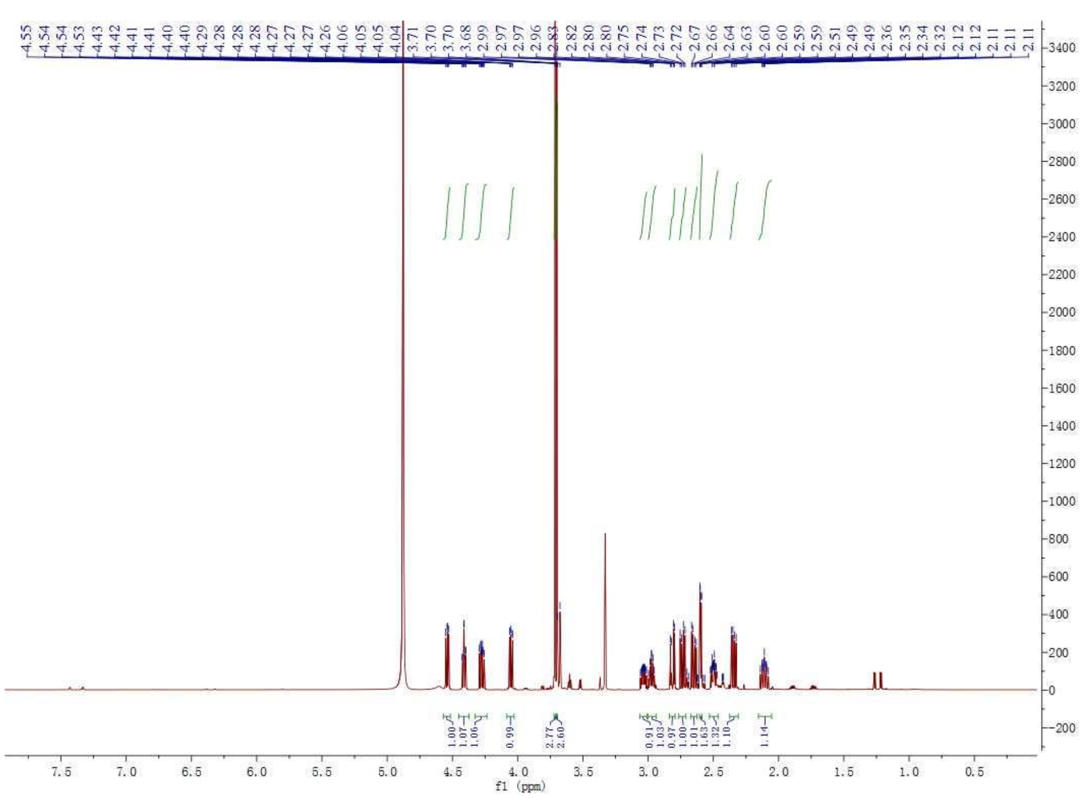

Figure S1. ^{1}H NMR spectra of **1** in CD_3OD

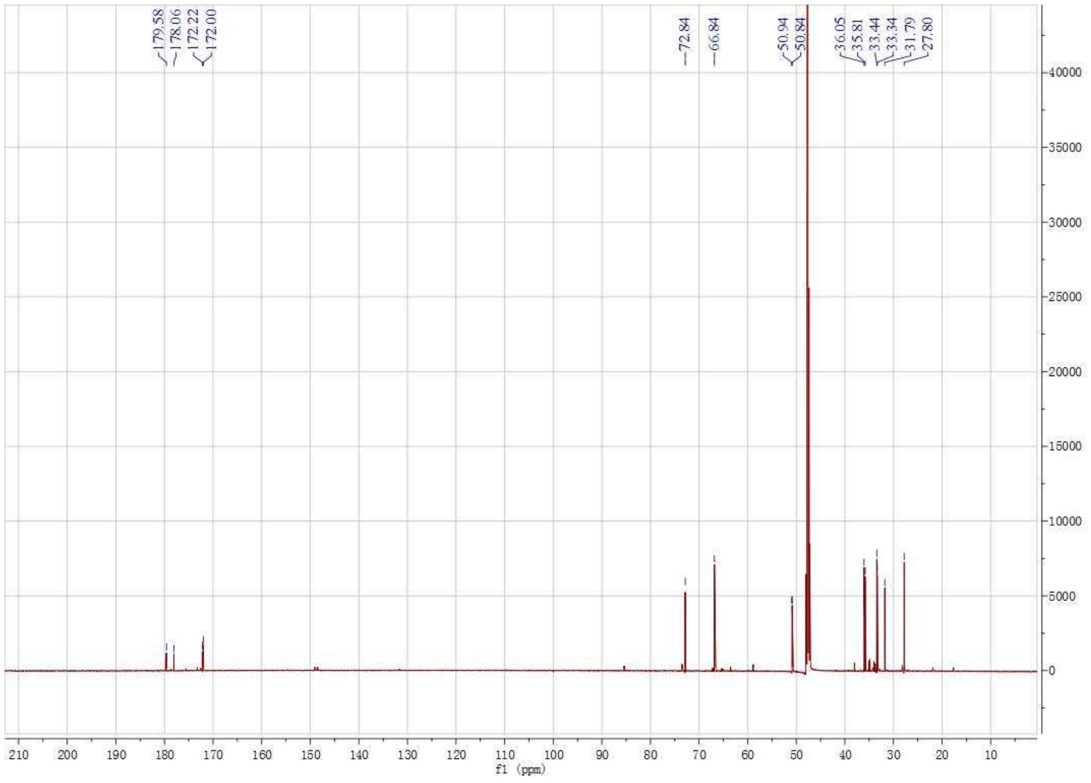

Figure S2. ^{13}C NMR spectra of **1** in CD_3OD

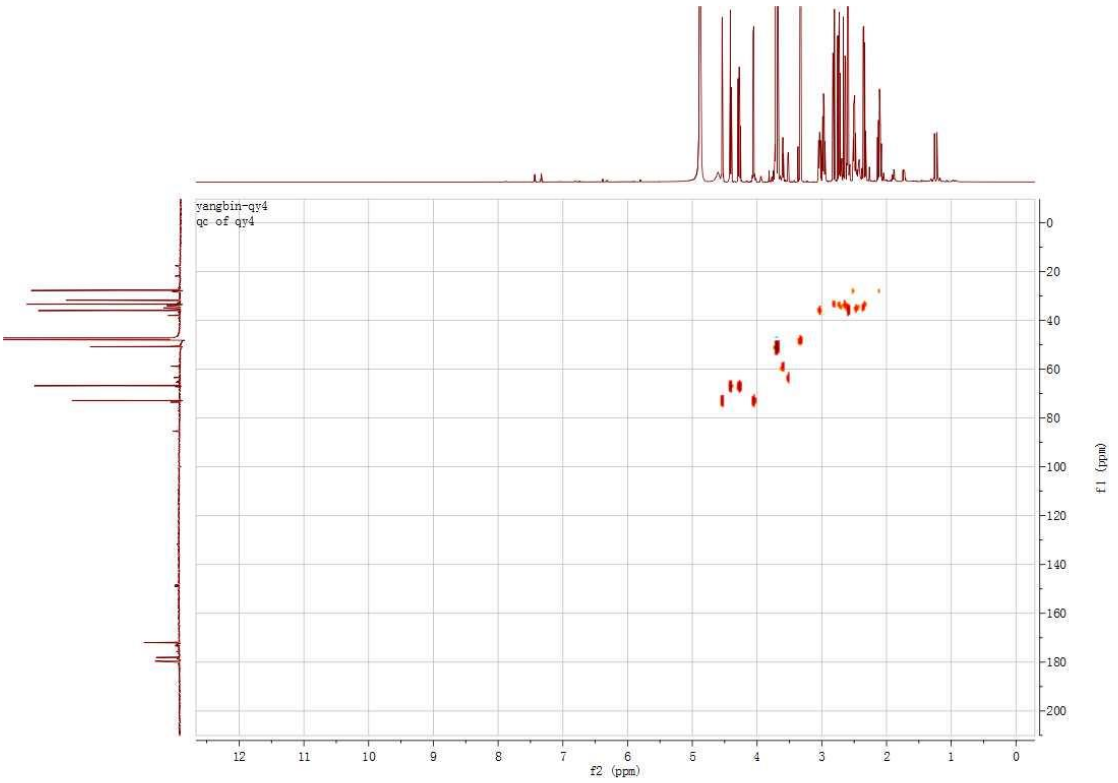

Figure S3. HSQC spectra of **1**

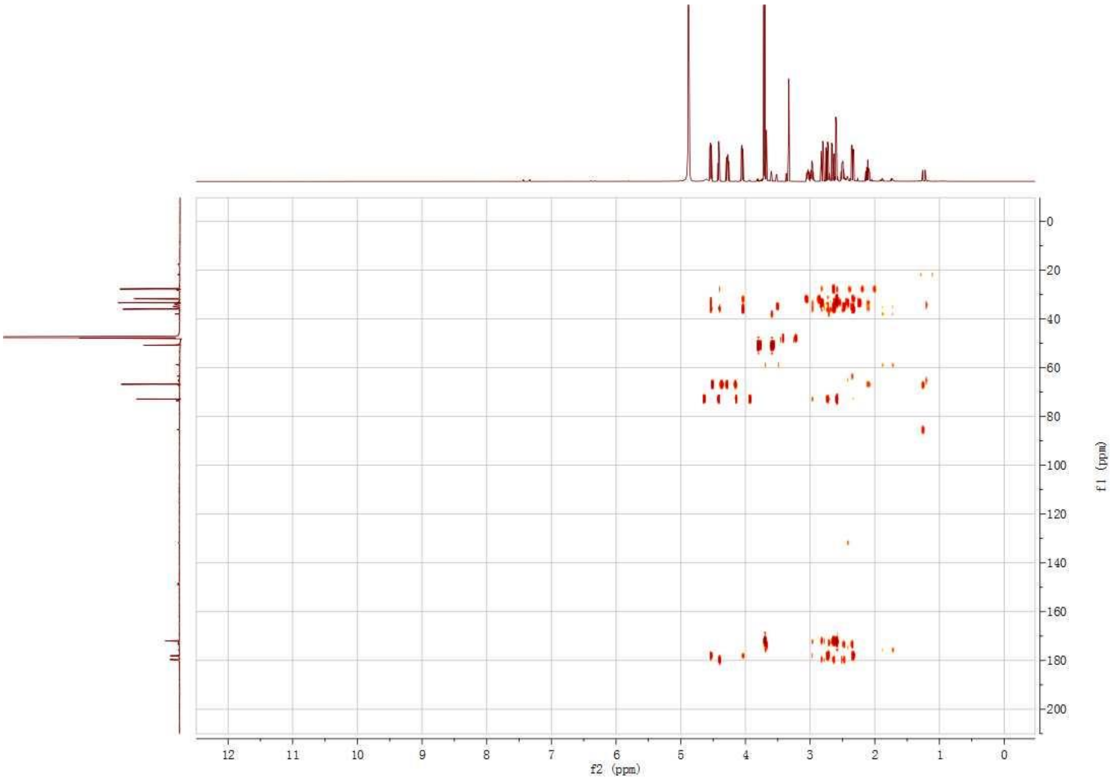

Figure S4. HMBC spectra of **1**

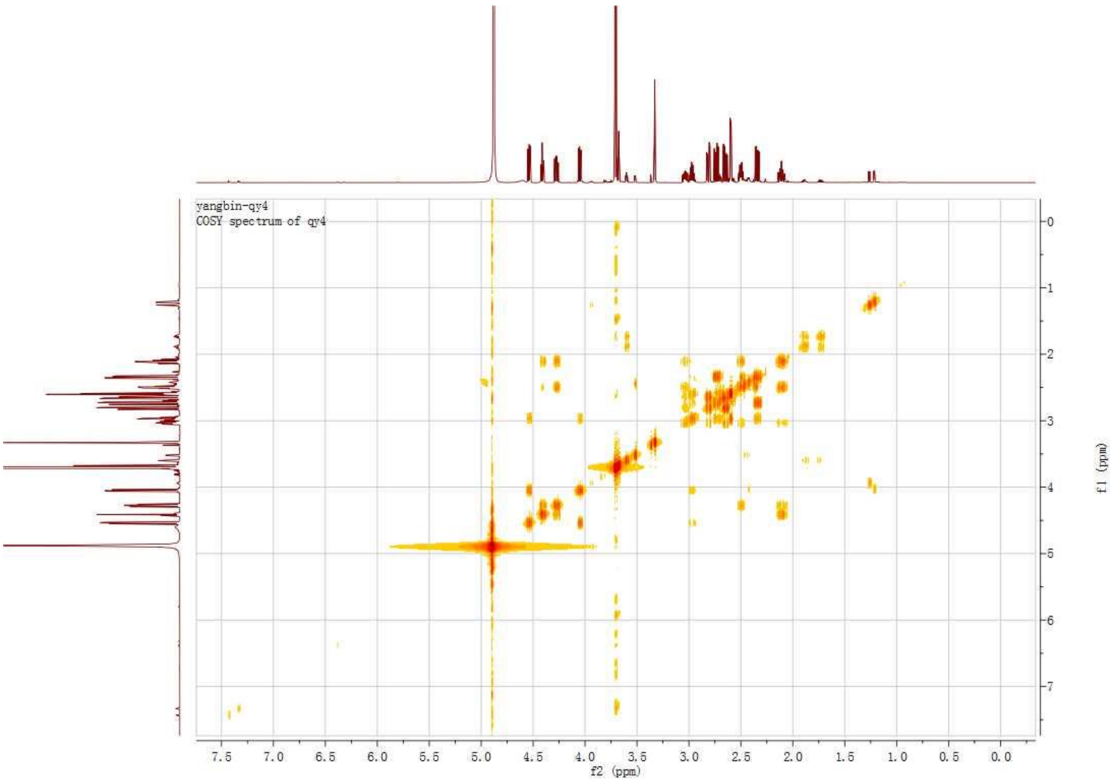

Figure S5. ^{1}H–^{1}H COSY spectrum of **1**

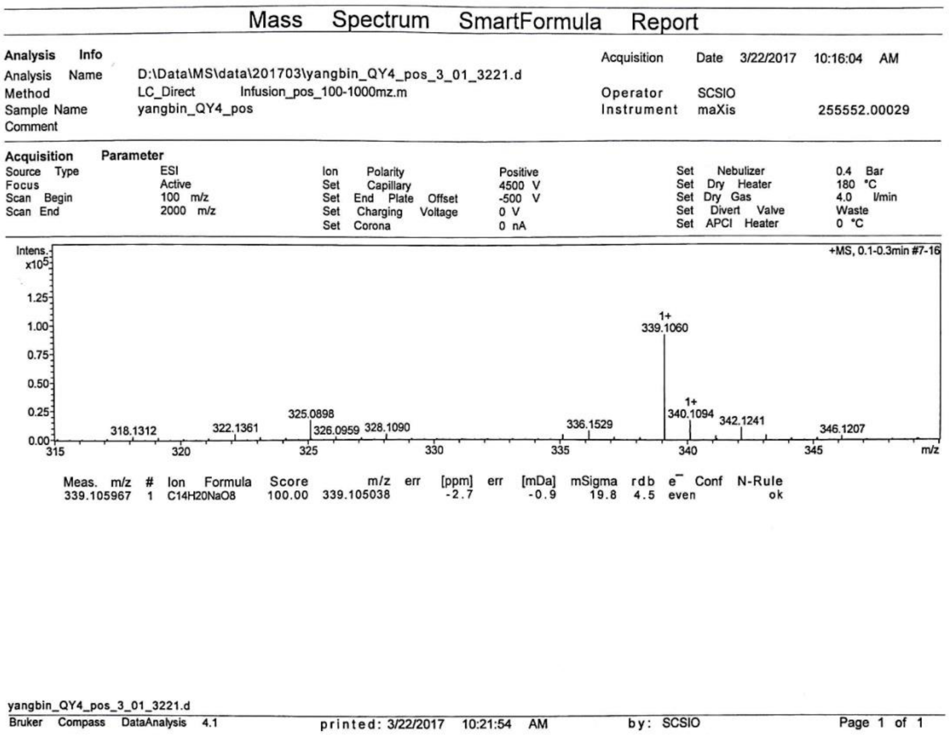

Figure S6. HRESIMS spectrum of **1**

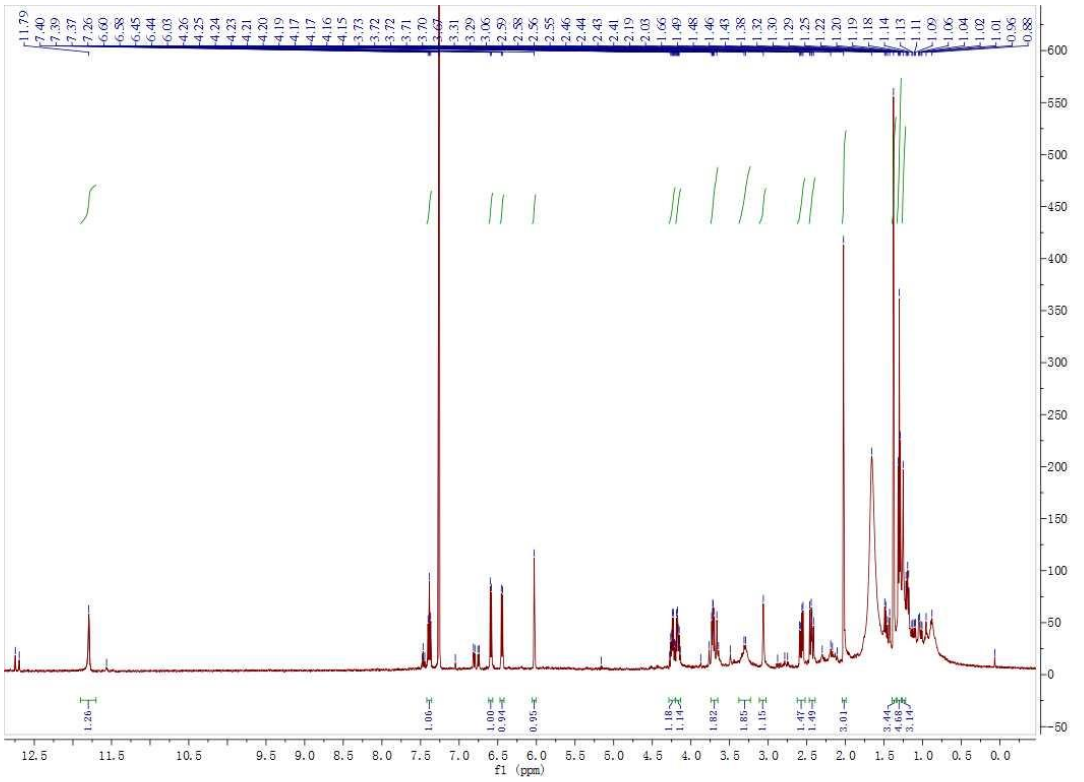

Figure S7. ^{1}H NMR spectra of **2** in CD_3OD

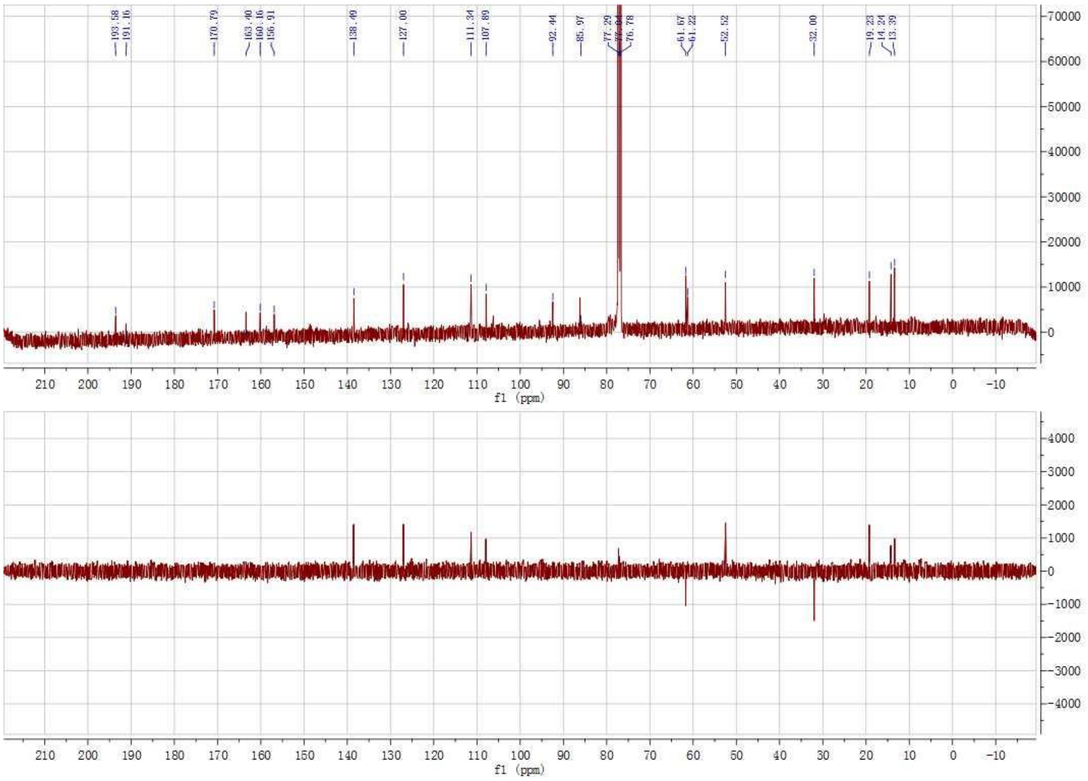

Figure S8. ^{13}C NMR spectra of **2** in CD_3OD

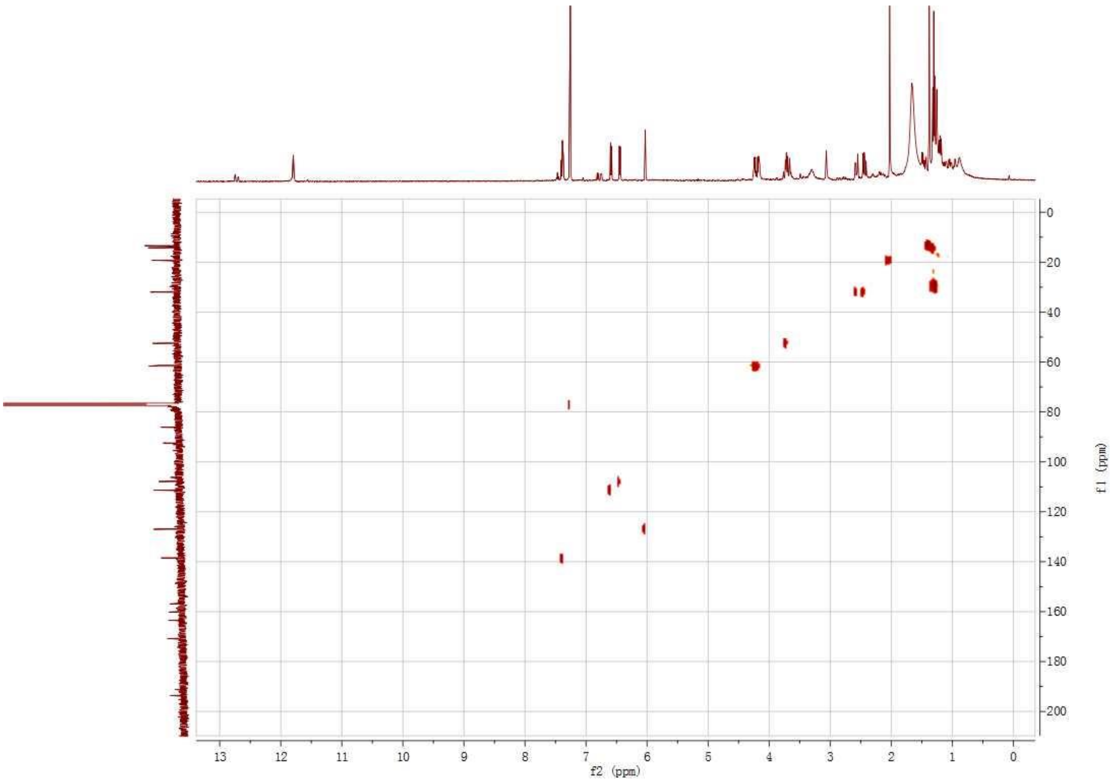

Figure S9. HSQC spectra of **2**

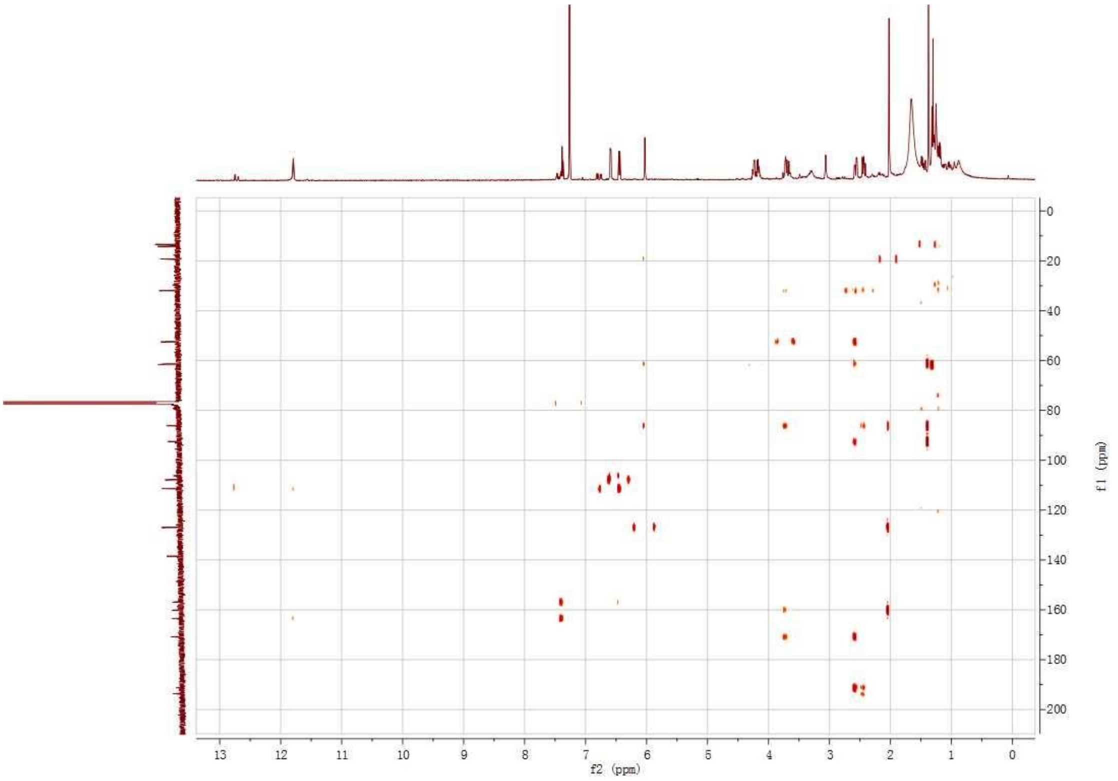

Figure S10. HMBC spectra of **2**

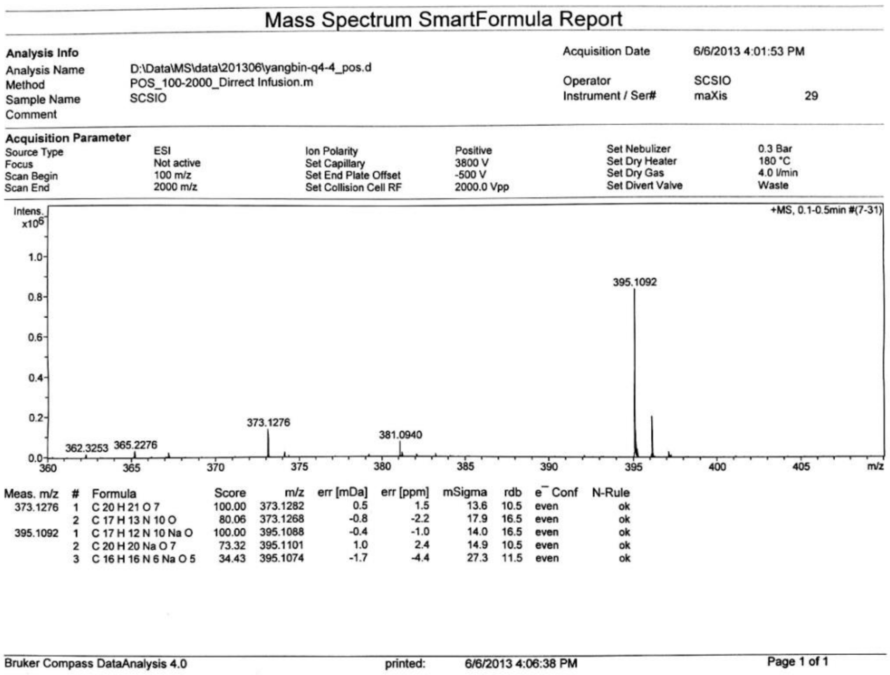
Mass Spectrum SmartFormula Report

Analysis Info
Analysis Name D:\Data\MS\data\201306\yangbin-q4-4_pos.d
Method POS_100-2000_Dirrect Infusion.m
Sample Name SCSIO
Comment
Acquisition Date 6/6/2013 4:01:53 PM
Operator SCSIO
Instrument / Ser# maXis 29

Acquisition Parameter

Source Type	ESI	Ion Polarity	Positive	Set Nebulizer	0.3 Bar
Focus	Not active	Set Capillary	3800 V	Set Dry Heater	180 °C
Scan Begin	100 m/z	Set End Plate Offset	-500 V	Set Dry Gas	4.0 l/min
Scan End	2000 m/z	Set Collision Cell RF	2000.0 Vpp	Set Divert Valve	Waste

Meas. m/z	#	Formula	Score	m/z	err [mDa]	err [ppm]	mSigma	rdb	e⁻ Conf	N-Rule
373.1276	1	C 20 H 21 O 7	100.00	373.1282	0.5	1.5	13.6	10.5	even	ok
	2	C 17 H 13 N 10 O	80.06	373.1268	-0.8	-2.2	17.9	16.5	even	ok
395.1092	1	C 17 H 12 N 10 Na O	100.00	395.1088	-0.4	-1.0	14.0	16.5	even	ok
	2	C 20 H 20 Na O 7	73.32	395.1101	1.0	2.4	14.9	10.5	even	ok
	3	C 16 H 16 N 6 Na O 5	34.43	395.1074	-1.7	-4.4	27.3	11.5	even	ok

Bruker Compass DataAnalysis 4.0 printed: 6/6/2013 4:06:38 PM Page 1 of 1

Figure S11. HRESIMS spectrum of **2**

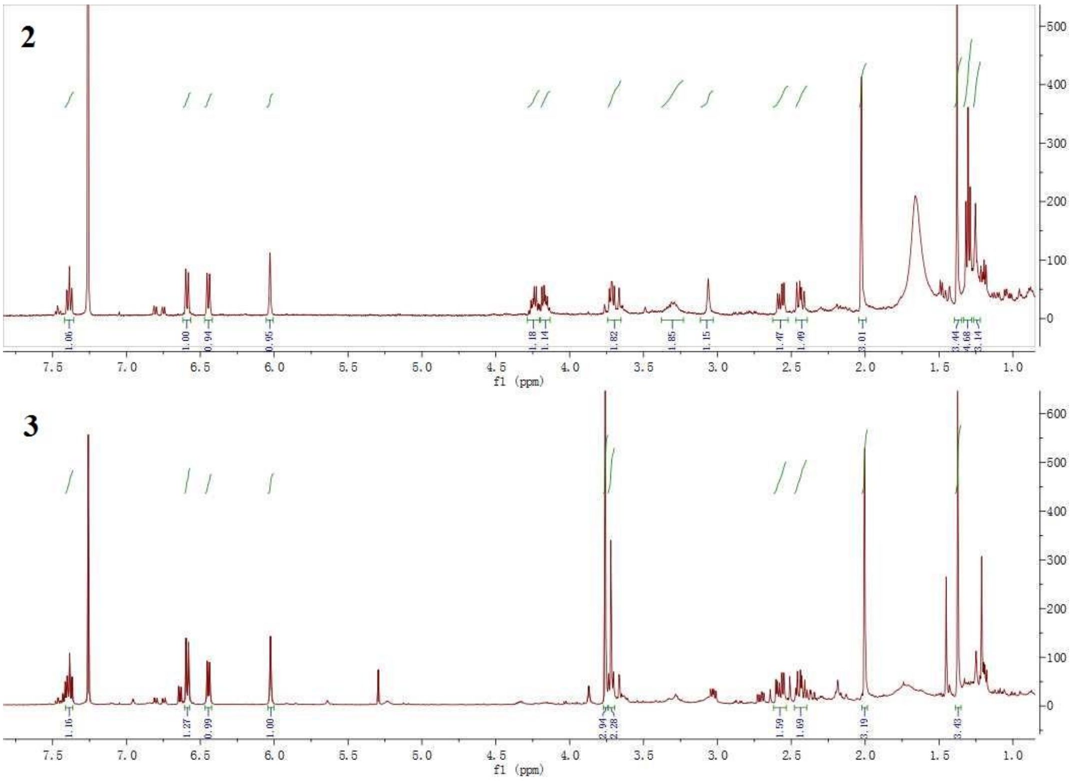

Figure S12. Compare ^{1}H NMR spectra between **2** and **3**

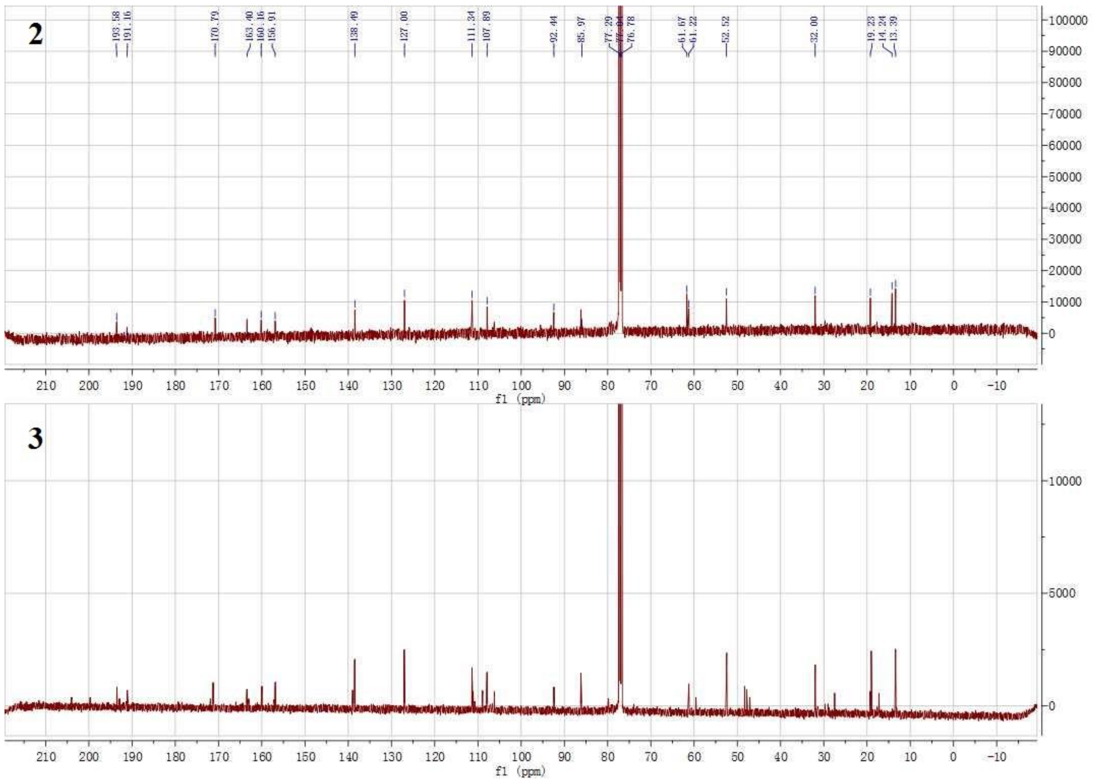

Figure S13. Compare ^{13}C NMR spectra between **2** and **3**

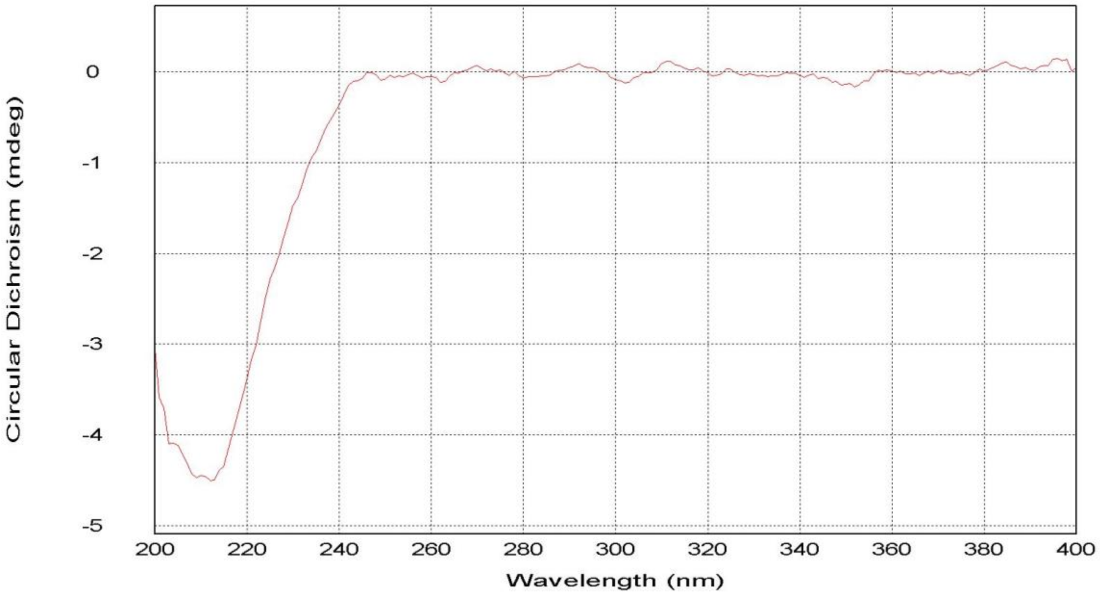

Figure S14. CD data of **1**

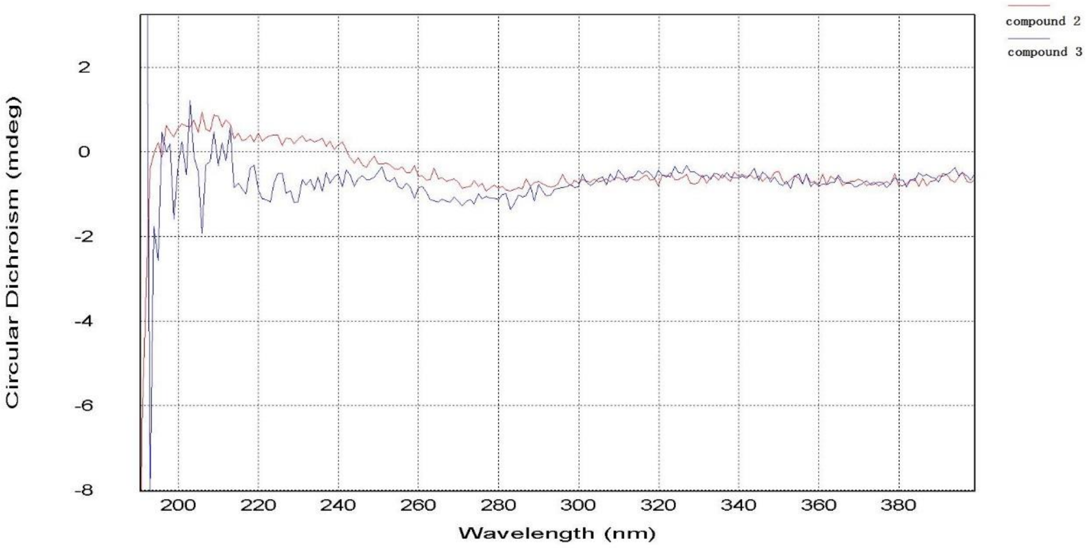

Figure S15. CD data of **2-3**
